# Dynamic Assessment of Plasma von Willebrand Factor and ADAMTS13 Predicts Mortality in Hospitalized Patients with SARS-CoV-2 Infection

**DOI:** 10.3390/jcm12227174

**Published:** 2023-11-19

**Authors:** Quan Zhang, Antonia Bignotti, Noritaka Yada, Zhan Ye, Szumam Liu, Zhe Han, X. Long Zheng

**Affiliations:** 1 Department of Pathology and Laboratory Medicine, The University of Kansas Medical Center, Kansas City, KS 66160, USA; 2 Department of Pulmonary and Critical Care Medicine, The Second Xiangya Hospital, Central South University, Changsha 410011, China; 3 Center for Precision Disease Modeling, Department of Medicine, University of Maryland School of Medicine, 670 West Baltimore Street, Baltimore, MD 21201, USA; 4 Institute of Reproductive and Developmental Sciences, The University of Kansas Medical Center, Kansas City, KS 66160, USA

**Keywords:** COVID-19, endothelial dysfunction, von Willebrand factor, ADAMTS13, mortality

## Abstract

Background: Plasma levels of von Willebrand factor (VWF) are significantly elevated in patients with coronavirus disease 2019 (COVID-19). However, dynamic changes and prognostic value of this biomarker in hospitalized patients with COVID-19 have not been determined. Methods: A total of 124 patients infected with SARS-CoV-2 were prospectively recruited for the study. Serial blood samples were obtained at the time of admission (D1), 3–4 days following standard-care treatments (D2), and 1–2 days prior to discharge or any time collected prior to death (D3). Plasma VWF antigen, ADAMTS13 antigen, and ADAMTS13 proteolytic activity, as well as the ratio of VWF/ADAMTS13 were determined, followed by various statistical analyses. Results: On admission, plasma levels of VWF in COVID-19 patients were significantly elevated compared with those in the healthy controls, but no statistical significance was detected among patients with different disease severity. Plasma ADAMTS13 activity but not its antigen levels were significantly lower in patients with severe or critical COVID-19 compared with that in other patient groups. Interestingly, the ratios of plasma VWF antigen to ADAMTS13 antigen were significantly higher in patients with severe or critical COVID-19 than in those with mild to moderate disease. More importantly, plasma levels of VWF and the ratios of VWF/ADAMTS13 were persistently elevated in patients with COVID-19 throughout hospitalization. Kaplan–Meier and Cox proportional hazard regression analyses demonstrated that an increased plasma level of VWF or ratio of VWF/ADAMTS13 at D2 and D3 was associated with an increased mortality rate. Conclusions: Persistent endotheliopathy, marked by the elevated levels of plasma VWF or VWF/ADAMTS13 ratio, is present in all hospitalized patients following SARS-CoV-2 infection, which is strongly associated with mortality.

## 1. Introduction

Coronavirus disease 2019 (COVID-19) [1], caused by severe acute respiratory syndrome coronavirus 2 (SARS-CoV-2), affected more than 600 million people globally. In the United States, more than 95 million cases of COVID-19, resulting in one million deaths, were reported. The overall mortality rate in patients with severe COVID-19 was 10–20%. The peak of reported cases in the U.S. was between February and March in 2022, reaching over 5 million cases per week. SARS-CoV-2 is reported to infect neuron cells, microglia, the epithelium, and macrophages via angiotensin-converting enzyme 2 (ACE2) [2,3]. Patients with COVID-19 may develop a mild “flu-like” symptom or severe acute lung disease, complicated by various thromboembolic diseases that require admission to hospital or an intensive care unit (ICU) for management. These patients are at risk of death despite prompt anticoagulant treatment [4].

Many studies have demonstrated that endothelial injury and microvascular thrombosis are associated with the pathogenic processes of severe and critical COVID-19 disease [5,6,7,8,9,10,11,12]. The vascular endothelium is a single and flat cell layer that maintains the entire balance of blood fluid hemostasis and prevents thrombosis [13,14]. Once the endothelial integrity is disrupted by certain exogenous or endogenous factors, vascular permeability and inflammatory processes develop. This results in shedding and degradation of endothelial glycocalyx, as well as the release of proteoglycan syndecan-1 [5,15], von Willebrand factor (VWF) [7], and thrombomodulin [16], etc. This sequence of events creates a conducive environment for the development of thrombotic episodes, particularly if the disease progresses and appropriate interventions are not provided.

VWF, a multimeric glycoprotein, is synthesized in the vascular endothelium and megakaryocytes. It is then stored in the Weibel–Palade bodies of endothelial cells or alpha-granules of megakaryocytes or platelets [17,18,19]. Upon stimulation by thrombin, mechanical shear, and inflammatory cytokines such as inteleukin-4 (IL-4) and tumor necrosis factor-α (TNF-α), large amount of VWF multimers may be released from endothelium. The released VWF multimers may remain anchored on the endothelial membrane [7,20] and form unusually large (UL) VWF bundles or strings that attract platelets and neutrophils to the sites of vascular injury [21,22].

These endothelium-bound ULVWF strings can be rapidly cleaved by a plasma metalloprotease ADAMTS13 (a disintegrin and metalloprotease with thrombospondin type 1 repeats 13) [23,24,25]. ADAMTS13 is primarily synthesized in hepatic stellate cells [26] and endothelial cells [27,28]. In contrast to the effect on VWF synthesis and release, most inflammatory cytokines, such as TNF-α, interferon-r, and inteleukin-6 (IL-6), appear to inhibit the synthesis of ADAMTS13 in hepatic stellate cells and endothelial cells [29,30,31]. The main function of plasma ADAMTS13 is to regulate hemostasis and inhibit inflammation through cleavage of VWF, thus preventing the excessive accumulation of VWF–platelet aggregates and neutrophils/monocytes [32,33]. A deficiency in plasma ADAMTS13 antigen or activity, resulting from inherited mutations in *ADAMTS13* [34] or acquired autoantibodies against ADAMTS13 [35,36], may lead to a potentially fatal blood disorder thrombotic thrombocytopenic purpura (TTP). However, mild to moderate reduction of plasma ADAMTS13 activity may be associated with an increased risk of many other inflammatory and thrombotic disorders, including ischemic cerebral infarction (or stroke) [37,38], myocardial ischemia or infarction [38,39], pregnancy-associated complications such as preeclampsia [40], trauma [41,42], and COVID-19- associated coagulopathy [43,44], etc.

However, the data published to date on the relationship between the VWF or VWF/ADAMTS13 ratios and the adverse events or complications of COVID-19 remain inconclusive [45,46,47,48,49,50,51], largely due to small sample sizes and single-time blood sampling on admission in previous studies. To better understand the role of VWF/ADAMTS13 axis in pathogenesis and prognosis of COVID-19, we determined the dynamic change and prognostic value of plasma levels of VWF and VWF/ADAMTS13 ratios in hospitalized patients with SARS-CoV-2 infection.

## 2. Materials and Methods

### 2.1. Patients and Sampling

The Institutional Review Board (IRB) of the University of Kansas Medical Center has approved the study (#00148313). A total of 124 consecutive hospitalized patients with a positive SARS-CoV-2 test by polymerase chain reaction (PCR) from January 2022 to March 2022 were prospectively recruited for the study (https://apps.who.int/iris/bitstream/handle/10665/330857/WHO-2019-nCoV-SurveillanceGuidance-2020.3-eng.pdf) (accessed on 7 October 2023), irrespective of their clinical signs and symptoms. The inclusion criteria for COVID-19 patients were: (1) age > 19 years old; and (2) hospitalized for COVID-19-related symptoms. For asymptomatic patient controls, we enrolled those admitted for a medical condition (such as prior to orthopedic surgery, depression, coronary artery disease, etc.) that was not primarily caused by COVID-19 but who tested positive for SARS-CoV-2 by PCR. Additionally, we included 23 local healthy individuals who were not acutely ill but matched for their age, gender, and race, as additional normal controls.

Citrated or ethylenediaminetetraacetic acid (EDTA)-anticoagulated blood samples were obtained on admission (D1), 3–4 days following treatment (D2), and 1–2 days prior to discharge or any time prior to death (D3). The exclusion criteria include those under 18 years old, and those who undergone surgery before an initial sample collection or those with an initial sample collected more than 48 h after the hospital admission. Patient demographic, clinical, and laboratory data are collected throughout the hospitalization and follow-up data are obtained from the electronic medical record. All patients are followed up for 60 days to obtain survival information.

### 2.2. Plasma VWF Antigen

An in-house enzyme-linked immunosorbent assay (ELISA) was used to determine the plasma VWF antigen level, as described previously [52]. Briefly, the wells of a 96-well plate were coated overnight with one rabbit anti-human VWF IgG (1:2000) from Agilent-Dako (Santa Clara, CA, USA). A pooled normal human plasma (NHP), defined as having 100% VWF (or 10 µg/mL), was used for a calibration. A total of 100 μL of serially diluted NHP or patient or control plasmas (1:100 dilution) were added to the wells and incubated for 2 h. Following an extensive wash with PBST (phosphate buffered saline with 0.05% tween 20 and 0.5% bovine serum albumin), another anti-VWF IgG conjugated with horseradish peroxidase (HRP) (1:5000) (Agilent-Dako) was added and incubated for one hour. Following wash, a chromogenic substrate was added for color reaction. The absorbance (450 nm) was measured in each well using a SpectraMax spectrophotometer (Molecular Devices, San Jose, CA, USA). The plasma VWF antigen level in patients and controls was expressed as the percentage normal in the NHP.

### 2.3. Plasma ADAMTS13 Antigen

The plasma ADAMTS13 antigen was determined by a commercially available ELISA kit according to the manufacturers’ instructions (R&D Systems, Minneapolis, MN, USA). Briefly, 50 µL of diluted (1:10) NHP, control samples, or EDTA-anticoagulated patient plasma samples (1:100 dilution) were added to the wells, precoated with anti-ADAMTS13 IgG, and incubated at room temperature on a horizontal orbital shaker (500 rpm). Following an extensive wash, a human anti-ADAMTS13 IgG conjugated with horseradish peroxidase was used to detect the bound ADAMTS13 antigen. Following the chromogenic substrate reaction, the absorbance at 450 nm was measured for each well using a SpectraMax spectrophotometer (Molecular Devices, San Jose, CA, USA). The intra- and inter-assay coefficients of variation (CVs) of this assay were also less than 8% and less than 10%, respectively.

### 2.4. Preparation of Recombinant VWF73 Peptide

The recombinant VWF73 was expressed in *E. coli* BL21 after transformation with a pQE-100-hVWF73 construct (containing a fragment from the VWF-A2 domain D1596-R1668 with 6xHis), as described previously [53]. The cells were grown in a Luna-Bertani (LB) broth supplemented with 100 µg/mL of ampicillin at 37 °C, at 225 rpm on a culture shaker until the optical density (OD) value of ~0.6 was reached. An isopropyl β-D-1-thiogalactopyranoside (IPTG) was added to induce VWF73 protein expression at 30 °C for 4–6 h. The cells were harvested by centrifugation at 3500 rpm at 4 °C for 20 min. The pellets were resuspended with a buffer containing a lysozyme to lyse the cells. After sonication of the cell lysate for 10 s, twice, the cell lysate was centrifuged at 12,000 rpm for 15 min at 4 °C. The supernatant was collected. Following the addition of 20 mM imidazole and pH adjustment to 8.0, the supernatant was loaded onto a nickel chelating column (Cytiva, Marlborough, MA, USA). The column was extensively washed with an equilibration buffer and disulfide bonds were reduced with a bond breaker (20 mM) for 15 min. The VWF73 peptide was labeled with a flurorescein-5-malemide (20 mM) in the dark for 15 min at room temperature. The labeled VWF-73 peptide was eluted with 250 mM imidazole, concentrated using a Millipore 3K centrifugal filter (Sigma-Aldrich, Burlington, MA, USA), and desalted and buffer-exchanged with 0.1% trifluoroacetic acid using a PD-10 column (GE Healthcare, Chicago, IL, USA). The concentration of fluorescein-labeled FRETS-hVWF73 was quantified spectroscopically by measuring the absorbance at 280 nm and 495 nm. Small aliquots were stored at −80 °C until use.

### 2.5. Plasma ADAMTS13 Activity

Plasma ADAMTS13 activity was determined by the FRETS-VWF73 assay described previously [53,54]. The cleavage of the recombinant fluorescein-labeled VWF73 substrate by plasma ADAMTS13 was performed at 25 °C in 5 mM Bis-Tris, 25 mM CaCl_2_, and 0.005% Tween 20, at pH 6.0 for one hour. The rate of fluorescence generation was determined every 2 min at 485 nm with an auto cutoff at 530 nm and 538 nm. A NHP was used for the calibration, which was defined as having 100 U/dL (or 100%) of ADAMTS13 activity.

### 2.6. Statistical Analysis

The means and standard deviations (SDs) were determined for continuous variables, but the medians and interquartile ranges (IQR) were determined for non-continuous variables. The Mann–Whitney U and Kruskal–Wallis tests were performed for comparison of 2 and ≥3 groups, respectively. Kaplan–Meier survival analysis with a log-rank test were used to determine the difference in survival rates of two groups. The Cox proportional hazard ratio regression determined the predictive value of these biomarkers at different sample collection time points for the mortality rate. All analyses were performed using either SPSS statistics version 26.0 (IBM, Armonk, NY, USA) or Prism 8.0 (GraphPad, Boston, MA, USA). The sample size was determined by a Shapiro–Wilk test.

## 3. Results

### 3.1. Demographic, Clinical, and Laboratory Characteristics of the COVID-19 Patient Cohort

A total of 124 hospitalized patients with SARS-CoV-2 infection and 23 healthy controls were included in the study. These SARS-CoV-2 infected patients were divided into four groups: asymptomatic, moderate, severe, and critical groups based on the updated WHO guidelines (WHO/2019-CoV/clinical/2021.2). Critical patients are defined by the presence of acute respiratory distress syndrome, sepsis, septic shock, etc.; severe patients are defined by any of the following: oxygen saturation < 90% on room air or respiratory rate > 30/min or signs of severe respiratory distress; moderate (non-severe) patients were defined as: absence of any criteria for severe and critical COVID-19. The demographic, clinical, and laboratory characteristics of this cohort of patients were previously described in a different study [5]. There was no significant difference in terms of demographic features, (e.g., gender, age, body mass index, and race) among various patient groups. A similar rate of comorbidity was found in these patients, including cardiovascular disease, diabetes mellitus, chronic obstructive pulmonary disease (COPD), chronic renal failure, hyperlipoidemia, cancer, and thromboembolic events except for hypertension.

### 3.2. Plasma Levels of VWF Antigen, ADAMTS13 Activity, and ADAMTS13 Antigen in Hospitalized Patients with SARS-CoV-2 Infection

**Figure 1:**
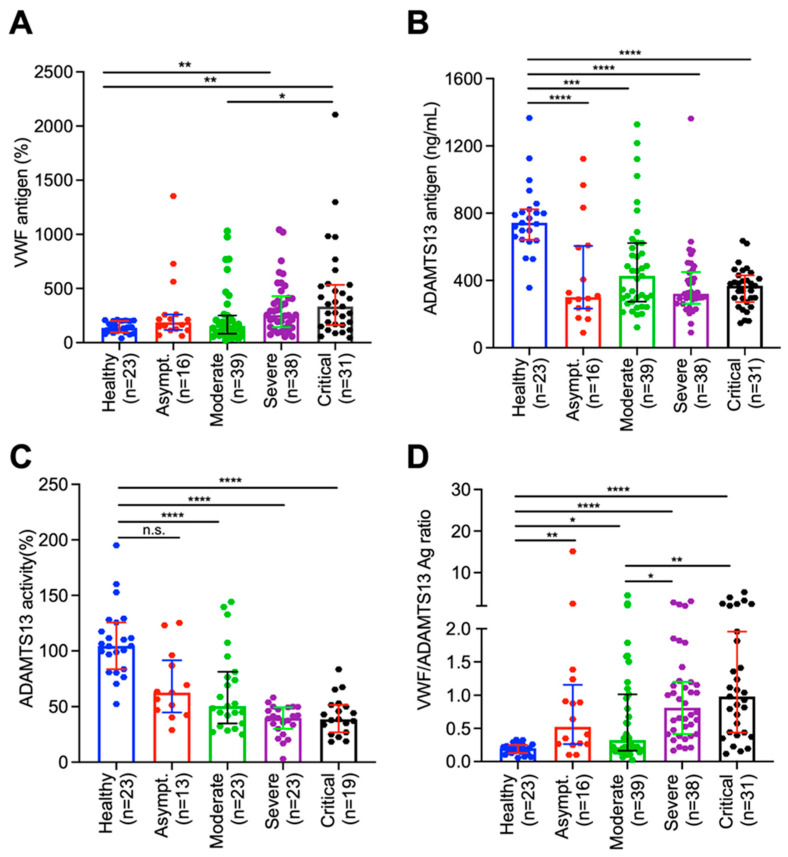
Plasma levels of biomarkers in hospitalized patients with SARS-CoV-2 infection and healthy controls. Admission plasma VWF antigen (**A**), ADAMTS13 activity (**B**), ADAMTS13 antigen (**C**), and the ratio of VWF/ADAMTS13 antigen in healthy controls, hospitalized patients with SARS-CoV-2 infection on admission (D1) with various disease severities (e.g., asymptomatic, moderate, severe, and critical) (**D**). The data shown are the median (bar) and interquartile range (IQR) (horizontal lines), as well as individual values [55]. Kruskal–Willis test was performed to determine the statistical significance of the difference among various groups. Here, n.s., *, **, *** and **** indicate a *p*-value >0.05, <0.05, <0.01, <0.005, and <0.001, respectively.

To determine the role of the VWF/ADAMTS13 axis in the pathogenesis of COVID-19 associated coagulopathy, we assessed the admission plasma levels of the VWF antigen and ADAMTS13 activity/antigen in COVID-19 patients with various disease severities. The median (IQR) admission levels of plasma VWF antigen in asymptomatic, moderate, severe, and critical groups were 136% (93–200%), 188% (120–259%), 153% (186–240%), 255% (143–411%), and 334% (165–526%), respectively. However, a statistically significant difference in the plasma levels of VWF antigen was only detected between the critical and moderate groups (*p* < 0.05), and between the severe/critical group and healthy controls (*p* < 0.01) (Figure 1A). Concomitantly, the admission plasma levels of the ADAMTS13 antigen (Figure 1B) and activity (Figure 1C) were significantly reduced in all hospitalized patients regardless of their disease severity (*p* < 0.005~0.001). More interestingly, the ratios (median, IQR) of plasma VWF/ADAMTS13 antigen in patients with asymptomatic (0.5, 0.3–1.2) (*p* < 0.01), moderate (0.3, 0.2–1.0) (*p* < 0.05), severe (0.8, 0.4–1.2) (*p* < 0.001), and critical (1.0, 0.4–2.0) (*p* < 0.001) COVID-19 were significantly higher than that in the healthy controls. Furthermore, the ratios of VWF/ADAMTS13 antigen in patients with severe/critical were significantly higher than those in patients with mild to moderate disease (*p*< 0.05~0.01) (Figure 1D).

### 3.3. Dynamic Changes in Plasma VWF and ADAMTS13 in Patients with SARS-CoV-2 Infection

**Figure 2:**
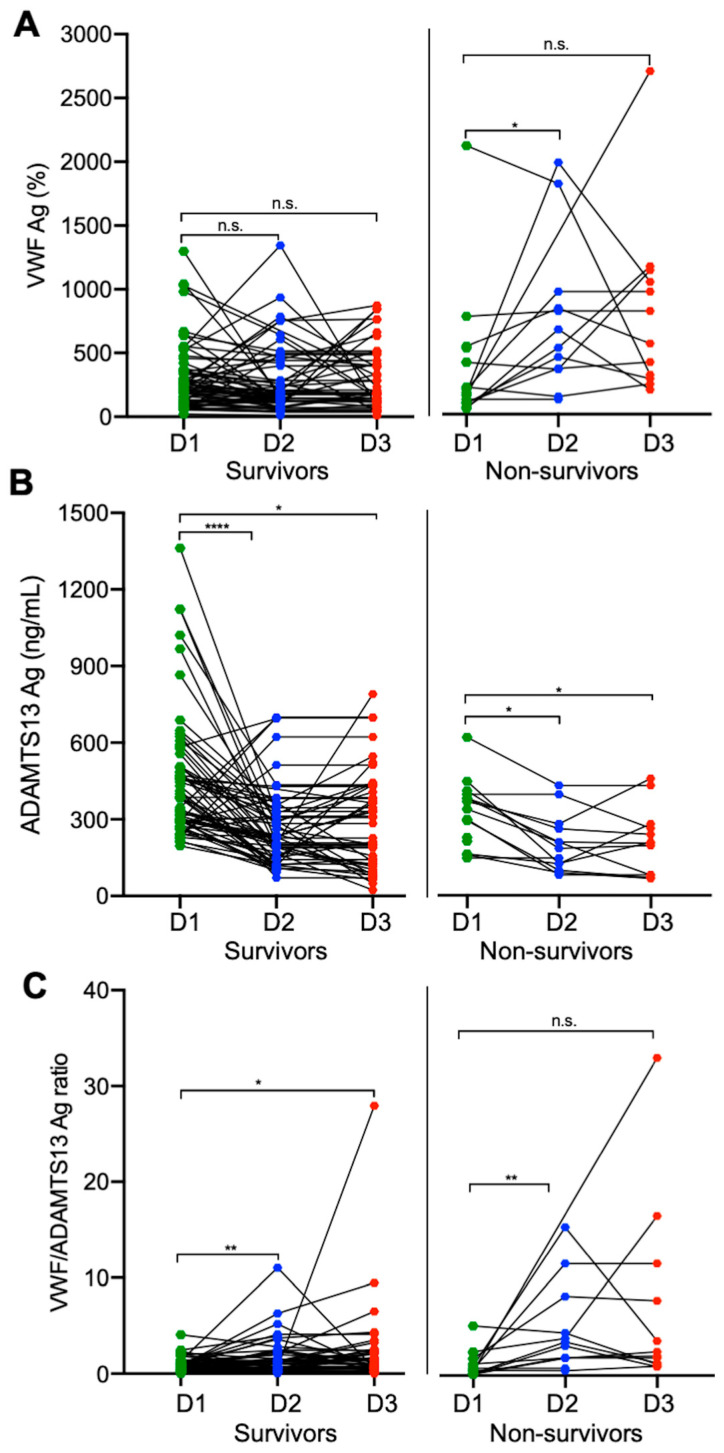
Longitudinal changes in plasma biomarkers in patients who survived and died of COVID-19. Each solid line depicts the change in plasma levels of VWF antigen (**A**), ADAMTS13 antigen (**B**), and the ratio of VWF/ADAMTS13 antigen (**C**) in patients who survived (survivors on the left) or died (non-survivors on the right) in each panel on admission (D1), 3–4 days following therapy (D2), and before discharge or death (D3). Data were analyzed by Wilcoxon test between two groups. Here, n.s., *, **, and **** indicate *p* > 0.05, <0.05, <0.01, and <0.001, respectively.

To assess the role of longitudinal changes in plasma VWF and VWF/ADAMTS13 ratios in predicting patient outcomes, we determined these biomarkers in serial blood samples of patients with severe and critical COVID-19. Our results showed that while there was significant individual variability in the plasma levels of VWF and ADAMTS13 antigen over time among all hospitalized patients infected with SARS-CoV-2, the plasma levels of the VWF antigen (Figure 2A) tended to increase with a concomitant decrease in the plasma ADAMTS13 antigen (Figure 2B) 3–5 days following treatment. This might be better presented by the ratios of VWF/ADAMTS13 antigen in Figure 2C. However, plasma VWF levels or the ratios of VWF/ADAMTS13 antigen in patients with severe/critical COVID-19 remained persistently higher than the normal over the entire hospitalization period, particularly in those who died (Figure 1A,D, right panels). These results suggest that persistent endotheliopathy in patients with severe/critical COVID-19 may be associated with an increased mortality rate.

### 3.4. Elevated VWF Levels or VWF/ADAMTS13 Antigen Ratios Predict 60-Day Mortality in Patients with Severe/Critical COVID-19

**Figure 3:**
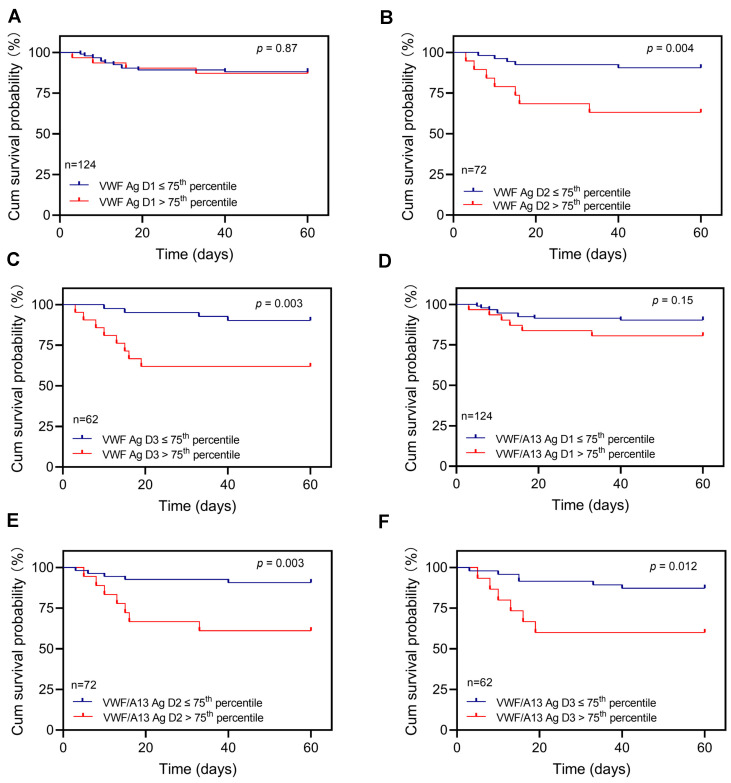
Kaplan–Meier survival analysis in patients with COVID-19 based on plasma levels of VWF or the VWF/ADAMTS13 ratio at different time points. The 60-day survival probabilities in patients with high (>75th percentile) and low (≤75th percentile) levels of plasma VWF antigen on admission (D1) (**A**), 3 to 4 days following therapy (D2) (**B**), and prior to being discharged or death (D3) (**C**) are shown. Similarly, the 60-day survival probabilities in patients with high (>75th percentile) and low (≤75th percentile) ratios of plasma VWF/ADAMTS13 antigen at D1 (**D**), D2 (**E**), and D3 (**F**) are also shown. Here, *p* < 0.05 and <0.01 are statistically significant and highly significant, respectively.

To further determine whether elevated plasma VWF levels or increased ratios of VWF/ADAMTS13 at various time points during hospitalization would predict long-term outcomes, we performed Kaplan–Meier survival and Cox proportional hazard risk analyses. Our results demonstrated that while the admission plasma levels of VWF had no or little predictive value (*p* > 0.05) (Figure 3A), patients with high plasma levels of VWF antigen (>75th percentile) at D2 (>459%) (*p* < 0.005) (Figure 3B) and D3 (>513%) (*p* < 0.005) (Figure 3C) had a dramatically higher 60-day mortality rate than those with low plasma levels of VWF antigen (≤75th percentile). Similarly, the elevated ratios of plasma VWF/ADAMTS13 antigen at D2 (1.98) (Figure 3E) and D3 (2.31) (Figure 3F), but not D1 (Figure 3D), also predicted the 60-day mortality rate.

**Figure 4:**
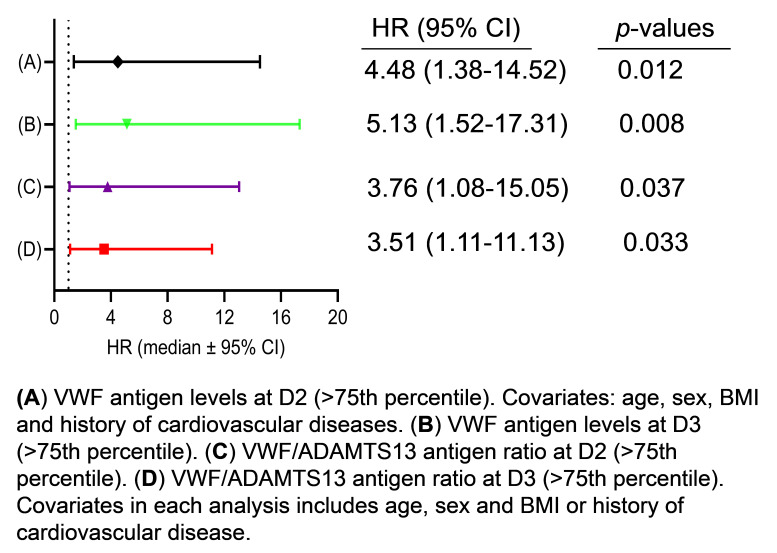
Cox proportional hazard regression analysis. The hazard ratios (HRs) for death were determined in patients with high VWF antigen (>75th percentile) 3–4 days following therapy (D2) (**A**) and prior to discharge or death (D3) (**B**). Additionally, the HRs were also determined in patients with high ratios of VWF/ADAMTS13 antigen (>75th percentile) at D2 (**C**) and D3 (**D**). The HRs were adjusted for covariates (age; sex; and body mass index, BMI), as indicated in each panel. Here, *p* < 0.05 and 0.01 are statistically significant and highly significant, respectively.

The Cox proportional hazard regression analysis revealed that the elevated plasma levels of VWF at D2 had a hazard ratio (HR) for mortality of 4.5 (95% CI, 0.38–14.52) (*p* < 0.05) after age, gender, body mass index, and cardiovascular disease were adjusted (Figure 4A). At D3, the elevated plasma VWF levels had an HR of 5.13 (95% CI, 1.52–17.31) (*p* < 0.01), also after age, gender, and body mass index were adjusted in the analysis (Figure 4B). Similarly, a predictive value was found with an increased ratio of plasma VWF/ADAMTS13 antigen at D2 (Figure 4C) and D3 (Figure 4D) for mortality; although, low levels of plasma ADAMTS13 antigen (<25th percentile) alone had no such a predictive value, regardless of the sampling time points (data not shown). These results indicate that the dynamic assessment of endothelial VWF or VWF/ADAMTS13 ratios may have a prognostic value for the long-term adverse outcome in patients with severe/critical COVID-19.

## 4. Discussion

The present study demonstrates that plasma levels of VWF antigen are significantly elevated in all hospitalized patients with SARS-CoV-2 infection regardless of their disease severity, and their median level of plasma VWF antigen increases by ~2 fold compared with that in healthy controls. Additionally, plasma levels of ADAMTS13 activity and antigen in these hospitalized and SARS-CoV-2-positive patients are significantly lower than those in healthy controls. Interestingly, only the plasma ADAMTS13 activity significantly reduced in patients with severe and critical COVID-19 compared with that in patients with mild to moderate disease. The elevated plasma levels of VWF and VWF/ADAMTS13 ratios persisted throughout hospitalization. Kaplan–Meier survival and Cox proportional hazard ratio analyses demonstrated a strong predictive value of the increased plasma levels of VWF and the ratios of VWF/ADAMTS13 for the 60-day mortality rate in patients with SARS-CoV-2 infection. Together, our results demonstrate that the elevated plasma levels of VWF or VWF/ADAMTS13 ratios predict the long-term mortality rate in hospitalized patients with COVID-19.

Early on in the COVID-19 outbreak, coagulopathy and fulminant thrombotic disorders emerged as a life-threatening complication in patients with severe and critical disease [56]. A meta-analysis showed that venous thromboembolism (VTE) occurred in 2.9% to 5.7% of all hospitalized COVID-19 patients, despite venous thromboprophylaxis with heparin or low-molecular-weight heparin [57]. Additionally, between 0.3% and 9.4% of patients were found to develop thrombosis after hospital discharge. These include both arterial and venous thromboses [58,59,60], suggesting the persistence of vasculopathy and coagulopathy in patients who had recovered from COVID-19. Omicron and other emerging subtypes of SARS-CoV-2 have become the dominant strains at the time of this study, and they appear to cause a relatively lower rate of hospitalization and fatality. However, 11.3% of our cohort of patients still experienced thrombotic events despite prophylactic anticoagulation and rigorous therapeutic intervention. To date, most prophylactic approaches target the secondary hemostasis by preventing the activation of the coagulation cascade [61]. Very few approaches are designed to target the primary hemostasis (e.g., VWF–platelet interaction), such as the use of aspirin, clopidogrel, and ticagrelor, etc. for prevention and treatment of COVID-19 associated coagulopathy [62,63].

The significant increase in plasma VWF levels in patients with COVID-19 may reflect the imbalance between the increased synthesis and release of VWF from the activated endothelium and a reduction in plasma ADAMTS13 activity or antigen. The reduction in plasma ADAMTS13 is largely due to the reduced biosynthesis and secretion following a SARS-CoV-2-induced cytokine storm [29,64]; although, a low titer of antibodies against ADAMTS13 was reported in 34.4% of patients with COVID-19, and this was higher (55.9%) in critically ill patients [65]. However, the individual variability of plasma VWF levels is large, which may be caused by the consumption of VWF multimers during thrombus formation. This may particularly be the case in patients with identifiable thromboembolic events, in whom the plasma levels of VWF may be significantly reduced instead of increased. The overall plasma levels of VWF antigen on admission in patients with thrombotic events remain significantly higher than those in the age-, gender-, and comorbidity-matched controls without thrombosis (data not shown). Thus, anticoagulation with unfractionated or low-molecular-weight heparin alone does not eliminate thrombosis in patients with severe and critical COVID-19. Other therapeutic modalities should be considered to eliminate the thrombotic complications in these patients.

Endotheliopathy, marked by significantly increased levels of plasma VWF antigen [66,67] and syndecan-1 [5], has recently been shown to be associated with COVID-19 disease severity. It may also predispose to long-term cardiovascular complications. The endotheliopathy may be caused by direct infection of the endothelium with SARS-CoV-2 through its binding to the outer membrane angiotensin-converting enzyme 2 (ACE2) receptor [68,69]; it may also be caused by shed viral nucleocapsid proteins [70] and spike protein [71,72], prolonged and overactive immune responses involving cytotoxic T cells and monocytes [73,74], and inflammatory cytokines including interleukin (IL)-4, IL-6, and tissue necrosis factor-α (TNF-α) [22,43]. It is known that these inflammatory cytokines trigger the release of VWF from endothelial cells while inhibiting the synthesis of ADAMTS13 in hepatic stellate cells and endothelial cells [29,75]. A marked increase in the plasma VWF antigen and a modest reduction in plasma ADAMTS13 antigen/activity may create a prothrombotic status, which may lead to microvascular thrombosis and tissue damage in major organs, such as the brain, heart, kidney, pancreas, adrenal glands, etc. This can then lead to long-term complications and mortality. Our results in this study are also in agreement with those reported previously from our laboratory. In a cohort of hospitalized patients with a suspected heparin-induced thrombocytopenia (HIT), high levels of plasma VWF antigen and low levels of ADAMTS13 activity were associated with increased mortality regardless of the HIT testing results by immunoassay or serotonin-releasing assay [76]. The prognostic value of the VWF/ADAMTS13 ratios in COVID-19 patients has also been reported by other groups [67,77]. Interestingly, plasma ADAMTS13 activity alone is not an independent predictor of all in-hospital mortality of COVID-19 patients after adjusting for other related factors in our study (not shown). This is consistent with the notion that the ratios of VWF/ADAMTS13 may play a crucial role in thrombus formation.

There are some limitations of our study. First, no data regarding plasma levels of VWF antigen and ADAMTS13 antigen/activity in these patients before SARS-CoV-2 infection or during the convalescent stage were available, which may complicate the data analysis in the longitudinal study. Second, we did not recruit asymptomatic outpatients as controls, although such a comparison has been previously made by others [78]. The hospitalized patients appeared to have higher levels of plasma VWF and lower levels of ADAMTS13 activity or antigen than the healthy controls, regardless of the reasons for being hospitalized. Third, this was a single-center study, and the sample size remained relatively small, particularly the follow-up samples, as the patient samples were collected every 2–3 days rather than daily. Some patients with mild to moderate disease may have been discharged home before we had a chance to collect the next samples.

To overcome these limitations, a large multi-center study with a prolonged follow-up may be necessary to confirm our findings and determine whether the ADAMTS13-VWF axis could help in the differential prediction of mortality in people with COVID-19. Moreover, even dynamic assessments could be useful in constructing laboratory-based machine-learning or decision tree models for rapid prediction in patients with severe/critical COVID-19, which has been implemented recently in differentiating COVID-19 from other viral infections [79,80].

Nevertheless, we can conclude that significantly elevated plasma levels of VWF antigen coupled with modestly reduced plasma levels of ADAMTS13 activity/antigen on admission and at other time points during hospitalization in patients with severe and critical COVID-19 may predict their adverse outcomes, such as 60-day mortality. These findings suggest the potential need for additional early prophylactic or therapeutic interventions targeting VWF or the VWF–platelet interaction, such as the use of caplacizumab, a newly approved drug used to treat TTP [81,82], or supplementation of recombinant ADAMTS13 [83,84] to improve endotheliopathy and reduce long-term outcomes in patients with severe and critical COVID-19.

## Data Availability

The datasets used and/or analyzed during the current study are available from the corresponding author on request.
